# Supplementary figures and images for: Influence of polydimethylsiloxane substrate stiffness on corneal epithelial cells
Source: R Soc Open Sci. 2019 Dec 4;6(12):191796. doi: 10.1098/rsos.191796 (PMC6936283; doi:10.1098/rsos.191796)

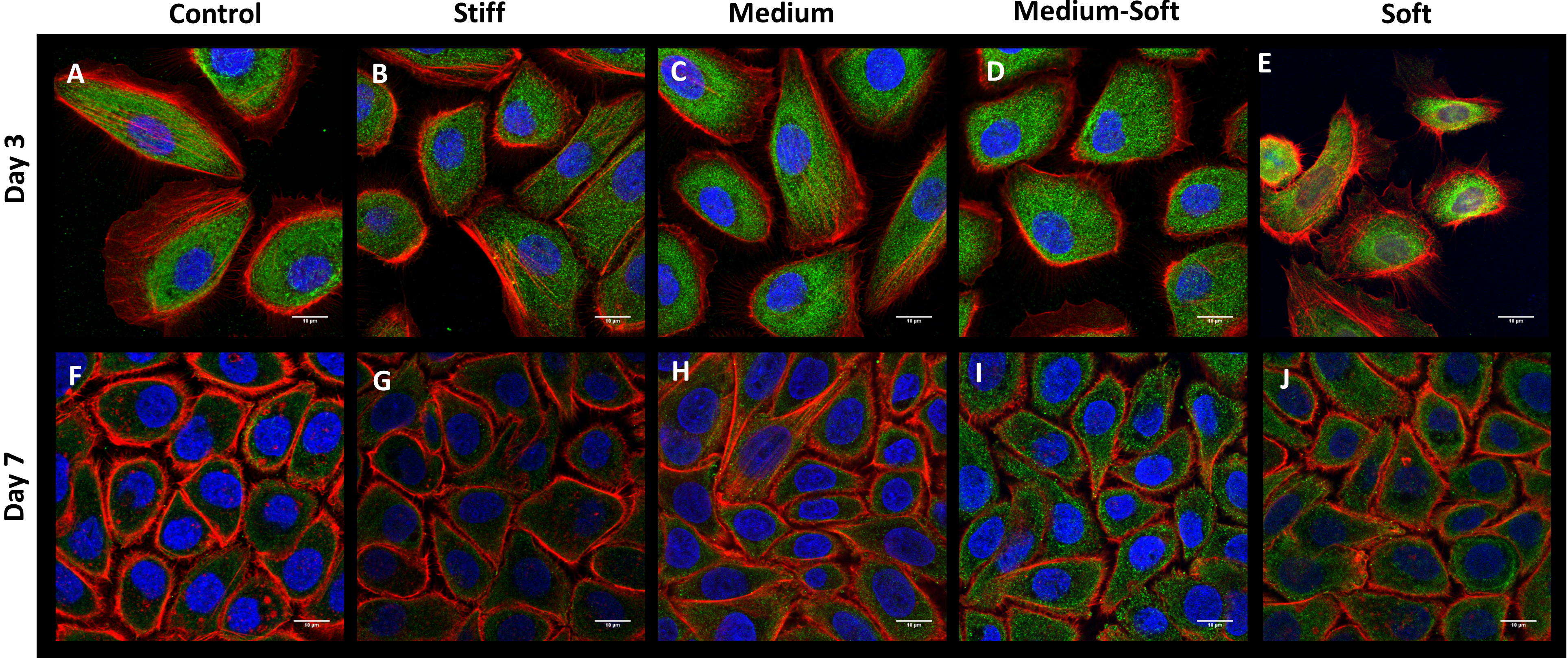

Supplement: Supplemental Figure 1 [file rsos191796supp1.tif]
